# Early adversity and prosocial behavior in adolescents from Bogotá: a cross-sectional study

**DOI:** 10.1186/s13034-024-00768-2

**Published:** 2024-07-08

**Authors:** Andrés Camilo Cardozo Alarcón, Nathaly Moreno Arenas, Kharen Alessandra Verjel Ávila, Elena María Trujillo Maza, Charlotte Greniez Rodríguez, Olga Lucía Vargas Riaño, Daniel Enrique Suárez Acevedo

**Affiliations:** 1 Universidad de los Andes, Ak. 7 #116-5, Bogotá, Colombia; 2 Alianza Educativa. Cl. 16 # 6-66, Bogotá, Colombia

**Keywords:** Early adversity, Well-being, Depression, Youth, Student, Mental health

## Abstract

**Background:**

The association between Adverse Childhood Experiences (ACEs), prosocial behavior, and depression (like other negative mental health outcomes) has not been thoroughly understood. This study aimed at evaluating their simultaneous association while controlling for key confounding variables.

**Methods:**

A cross-sectional study was carried-out with 2918 secondary school students from seven charter schools located in low-resourced neighborhoods in Bogota (Colombia), 54.12% were female, and mean age was 13.81 years. The self-report instrument included demographic variables, well-being, mental health, risk behaviors and symptoms of psychopathology. Assessment of ACEs was done by a series of yes/no questions, prosocial behavior was evaluated with the corresponding subscale in the Strengths and Difficulties Questionnaire, and depression was assessed with the Self-Reporting Questionnaire. Associations were tested using the Spearman correlation coefficient, Z tests and Chi-square tests, and all primary outcome analyses were adjusted for potential confounding variables through multivariate logistic regression using depression as outcome.

**Results:**

Mean exposure to ACEs was 3.15 events; those exposed to four or more obtained lower scores in well-being, satisfaction with life and family functioning, and higher scores in symptoms of psychopathology. For the prosocial behavior scores, 64.35% were classified as close to the average, 17.51% as slightly lowered, 11.91% as low, and 6.23% as very low; participants with higher levels of prosocial behavior showed lower scores in symptoms of psychopathology. While ACEs had a positive association with depressive symptoms (Odds Ratio [OR] 2.21, 95% confidence interval [CI] 1.67–2.94), prosocial behavior did not have a significant association with either ACEs or depressive symptoms in multivariate regression models.

**Conclusions:**

Novel studies should further elucidate the developmental pathways involving positive and negative mental health constructs to better understand the actual effectiveness of interventions that use these constructs in their design.

**Supplementary Information:**

The online version contains supplementary material available at 10.1186/s13034-024-00768-2.

## Background

Adverse Childhood Experiences (ACEs) are a cluster of potentially traumatic events that occur in childhood and adolescence [1, 2]. ACEs can be defined as “those experiences which require significant adaptation by the developing child in terms of psychological, social and neurodevelopmental systems, and which are outside of the normal expected environment” [3]. They include exposure to several forms of abuse and neglect, such as physical violence or emotional neglect, and some forms of household dysfunction, such as substance misuse and divorce, among others [1, 4]. ACEs are associated with risky behaviors, transmissible and non-transmissible diseases, injuries, and mental health issues, through direct and indirect pathways [5]. Regarding mental health illness, ACEs are associated with depressive symptoms, drug use, and conduct disorders, among others [6, 7].

ACEs affect a high proportion of the population. According to a retrospective study in twenty-three states of the United States, 61.55% of adults reported at least 1 ACE and 24.64% reported 3 or more ACEs [8]. In Latin America and the Caribbean, a study of violence against children found a prevalence of 30–60% for physical and emotional violence by caregivers, 17–61% for physical violence by students and 60 to 92% for emotional violence [9]. Similarly, in Colombia, an upper middle-income country from Latin America, it was found that adults have a prevalence of 64% for at least one ACE and 23% for four or more ACEs [10], and 29.3% of adolescents have suffered at least one traumatic event [11]. As for mental health outcomes, the most frequent illness related to ACEs is depression, with a risk increase of 2.7 times in women and 2.5 in men in comparison to those who have not been exposed [12]. Other associated mental health outcomes are use of psychotropic medication, tobacco dependence, and post-traumatic stress disorder [6, 13].

The developmental pathway from early adversity to depression has not been understood thoroughly yet, and it has not been either for many other outcomes. To account for the difference in long-term outcomes of people exposed to high levels of adversity, it is especially important to understand how people with exposure to early adversity differ in terms of protective factors. There are many ways trauma can influence the development of mental disorders, and its association with prosocial behavior has not been looked at in depth. ACEs may delay the development of prosocial behavior, increasing psychosocial adjustment problems [14]. For example, young adults who experienced childhood or adolescent maltreatment are more likely to exhibit psychopathic traits and less altruistic attitudes [15], and physical abuse is a positive predictor of deviant behaviors [16]. Besides, some evidence suggests that childhood adversity can lead to prosocial behaviors in adulthood through motivations like enhanced empathy, attempts to ameliorate the consequences of ACEs, finding a sense of purpose, and helping others with similar experiences [17].

Prosocial behavior is the action of empathy and responsibility for the well-being and needs of others, it can be expressed as caring, volunteering or helping others [18, 19]. The evidence reveals that acting in a prosocial and altruistic manner could increase someone’s self-concept [18], and a good self-concept in early adolescence will lead to better decisions during the transition to adulthood [20]. Further, elevated levels of prosocial behavior have been associated with better peer acceptance and academic achievement [21], have been reciprocally and directly associated with a positive affect [22], and negatively associated with both aggression and delinquency, even in early and late adolescence [23]. Moreover, a negative association between prosocial behavior and depressive symptoms has been reported in adolescents [24], however, adolescents with major depressive disorder seem to have increased prosocial behavior, which might be influenced by gender, relief from negative emotions or social-evaluative concern [24].

The evidence described above shows why it is needed to better understand the direct and indirect pathways leading to a major mental health outcome, such as depression. Both ACEs and prosocial behavior are associated with negative and positive outcomes regarding mental health and well-being in adulthood and adolescence. Nonetheless, the interaction between these variables and their role as protective or risk factors for the mental health of adolescent population is not well understood. Particularly, depression is associated with ACEs but there are mixed results regarding prosocial behavior, thus, understanding these paths could lead to the development of prevention strategies. Therefore, in the present study, we aimed to identify the relationship between ACEs, prosocial behavior and depression in adolescents from urban schools in low-resourced neighborhoods from Bogota, Colombia. We hypothesized that (1) ACEs had a positive association with depressive symptoms, (2) prosocial behavior had a negative association with both ACEs and depressive symptoms, and (3) prosocial behavior could modify the association between ACEs and depression after controlling for other variables.

## Methods

A cross-sectional study was carried out through a virtual, anonymous, self-administered survey with secondary school students from seven charter schools located in low-resourced neighborhoods in Bogota (Colombia). The locations of the target population are characterized by being hotspots of social issues including crime, significant levels of domestic violence and poverty. The net coverage rate in secondary education is 71% and 41.4% in high school, and there are families who have been victims of internal displacement [25]. For over 15 years, our research team had worked on several projects with the alliance of charter schools involved in this survey, and this specific project had a higher degree of community participation. The schools were involved in all stages of research, from the design of the questionnaire to the consent process and publication (CGR and OLVR were school staff at the time).

The project was reviewed and approved by the Research Ethics Committee at the School of Medicine, Universidad de los Andes, Colombia (N20190726). Legal guardians were given information on the project at school meetings, and those willing to participate gave their written informed consent. The adolescents gave their assent to participate after their legal guardians had given their consent. Students were informed that their participation was voluntary, that answering or not answering had no academic repercussions, and that they could skip questions or finish the questionnaire when they wished to. As the questionnaire would be completely anonymous, the students were given written and verbal information on how to get help from the schools and the research team, in case they felt distressed. Besides, in Colombia, it is mandatory for the principal investigators to leave their personal information contact (telephone and e-mail) on the consent form for the legal guardians.

At the time of recruitment (second term, 2019), there were a total of 3710 secondary school students who were eligible to participate, and we estimated that at least 600 students were needed to detect differences of 5% points in the prevalence of the outcomes of different groups with a level of statistical significance of 5% in two-tailed hypothesis tests.

There were no exclusion criteria and all secondary school students were invited to participate. A total of 2918 adolescents participated (response rate of 77.45%), and 1576 of these were female (54.12%). 2724 students reported their age, with a mean of 13.81 years (Standard Deviation [SD] 1.82, Interquartile Range [IQR] 12–15). 2910 participants reported their school grade, 607 (20.86%) were in 6th grade, 598 (20.55%) in 7th grade, 483 (16.6%) in 8th grade, 410 (14.09%) in 9th grade, 482 (16.56%) in 10th grade, and 330 (11.34%) in 11th grade.

The questionnaire included screening instruments freely available and validated in Spanish, and many have been used in Colombia by other authors and by our team in some of the participant schools in previous research projects. They measure well-being, mental health, risk behaviors and symptoms of psychopathology, and were selected because the schools wanted to have diverse array of variables to monitor in their institutions afterwards, and our team wanted the questionnaires to be easily comparable with the latest National Mental Health Survey, as well as to be able to control for confounding variables when analyzing the data [26]. The questions that do not belong to a specific instrument and the selected instruments were reviewed by the researchers and members of the schools to ensure their acceptability for the participants, and the survey was administered in the computer rooms of the educational institutions. To ensure data validity and confidentiality, the survey process was supervised by teachers and counselors who had participated in the research project design, had worked with us to anticipate issues in the questionnaire, and allocated time for the computer rooms to be under their control with not too many students. Demographic variables included were age, sex, school, and education grade at enrollment (sixth to eleventh grades in our education system).

After reviewing instruments available elsewhere and in Colombia [27, 28], the team decided to conduct the assessment of ACEs by a series of customized yes/no questions about 19 different experiences (see Box 1 in Additional file 1), so a total score could be summed up from 0 to 19 points, and we considered 4 or more as the high-risk category. Prosocial behavior was evaluated with the Strengths and Difficulties Questionnaire (SDQ)—Prosocial subscale [29]. The prosocial score was calculated using five items (e.g., *I am helpful if someone is hurt, upset or feeling ill*), 0–2 points per item (*not true, somewhat true or certainly true*), and ranked as follows: 7–10, close to the average; 6, slightly lowered; 5, low and 0–4, very low [30]. We used the cut-offs available from the authors because there are not published cut-offs in Colombia, and we have found that the authors’ guidelines have clinical value in the projects we have used this instrument before.

Other instruments and variables assessed in this study were:

- World Health Organization-Five Well-Being Index (WHO-5): A five-item questionnaire (*e.g., over the last two weeks, I have felt cheerful and in good spirits?*) using a 6-point Likert scale (from *‘all of the time’* to *‘at no time’*), 0–5 points per item, with total scores ranging from 0 to 25. A total score of < 13 or a score ≤ 1 in any item indicates poor well-being [27]. We used the Spanish translation of the questionnaire that is freely available from the World Health Organization Regional Office for Europe [31], versions with a 4-point Likert scale have been studied in Colombian samples with acceptable overall psychometric performance [32], and the adult version of the scale has been suitable for children and adolescents [33].
- Satisfaction With Life Scale (SWLS): A five-item questionnaire (e.g. *In most ways my life is close to my ideal*), using a 7-point Likert scale (from ‘*Strongly agree’ to‘Strongly disagree’*), 1–7 points per item, with total scores ranging from 5 to 35. Ranked as follows: 31–35, very satisfied; 26–30, satisfied; 21–25, a little satisfied, 20, neutral, 15–19, a little dissatisfied, 10–14, dissatisfied, and 5–9, very dissatisfied [34, 35].
- Family Adaptability, Partnership, Growth, Affection, and Resolve (Family APGAR): A five-item questionnaire (e.g., *I find that my family accepts my wishes to take on new activities or make changes in my life-style*) using a 5-point Likert scale ( from ‘*almost always’ to ‘hardly ever’*) (0–4 points per item), with total scores ranging from 0 to 20. Ranked as follows: 0–9 indicates severe dysfunction; 10–13, moderate dysfunction; 14–17, mild dysfunction, and ≥ 18 is considered good functioning [36, 37].
- Strengths and Difficulties Questionnaire (SDQ)—Difficulties scores [29, 30]: The 20-item self-report SDQ items were divided between 4 subscales of 5 items each (0–2 points per item): Hyperactivity (e.g., *I am restless, I cannot stay still for long)*, Emotional Symptoms (e.g., *I have many fears, I am easily scared*), Conduct Problems (e.g., *I take things that are not mine from home, school or elsewhere*), and Peer Problems (e.g., *Other children or young people pick on me or bully me*). Each item score from 0 to 2 (*not true, somewhat true, or certainly true*), each subscale has a score ranging from 0 to 10, and all scores are summed up to generate a total difficulties score from 0 to 40. The scores for total difficulties were ranked as: close to the average (0–14 points), 15 to 17 slightly raised (15–17 points), high (18–19 points), and very high (20–40 points). We used the cut-offs available from the authors as was already stated for the SDQ – Prosocial subscale.
- Self-reporting questionnaire, 20-item version (SRQ-20) [38]: A 20-item questionnaire using a yes/no format, with possible total scores ranging from 0 to 20 (positive screen ≥ 8), as well as an anxiety subscale score (0–10 points, positive screen ≥ 5) and a depression subscale score (0–13 points, positive screen ≥ 7). Some example items are: ‘*Do you sleep badly?’, ‘Are you easily tired?’, ‘Do you feel unhappy?’.* This is a widely used screening instrument for anxiety/depressive disorders, and the present study adopted the cut-off points used in the Colombian National Mental Health Survey (NMHS) 2015 [26, 39].
- Self-injurious behavior: lifetime prevalence of non-suicidal and suicidal types, and number of episodes per type (once, 2–5 times, 6 or more times).

A STATA® database was generated, and the quality and veracity of the information was checked. Most variables had complete data for more than 95% of the sample, and therefore missing values were excluded from each analysis. Central tendency and dispersion measures, as well as graphical inspection of the data, were used to detect possible extreme values and unexpected distributions. Internal consistency of the scales was evaluated using Cronbach’s alpha.

Bivariate association was tested using the Spearman correlation coefficient, Z tests and Chi-square tests, according to the characteristics of each variable. The main bivariate association shown in this manuscript is the Spearman correlation coefficient between ACEs and prosocial behavior. These bivariate analyses were used to find what variables could be potential confounders as they are simultaneously associated with depression and either ACEs or prosocial behavior. To reduce bias, all multivariate association analyses were adjusted for potential confounding variables through logistic regression, and the possibility of multicollinearity was ruled out using Variance Inflation Factor (VIF) analyses and conducting additional regression models excluding variables that could duplicate questions. We used depression as the outcome, ACEs and prosocial behavior as independent variables, and include demographics and the rest of the mental health variables as covariates to ascertain whether the association between the outcome and independent variables was significant in the presence of other associations. Crude and adjusted Odds Ratios (OR) were obtained in these regression models, and the interaction term for ACEs and prosocial behavior was tested separately as well.

## Results

Table S1 (Additional file 1) displays the distribution of all mental health indicators for the whole sample, with internal consistency for total scores and subscales. All scales and subscales used had a good internal consistency, except some of the SDQ subscales. Only 361 respondents reported no exposure to ACEs, 2557 (87.63%) reported one or more, and 1087 (37.25%) reported four or more ACEs. On average, participants reported being exposed to 3.15 ACEs (SD: 2.55, median: 3, IQR: 1–5). For all ACEs, missing data was less than 5%, except for the question on partner violence as 1038 (35.57%) have not had a partner ever. As shown in Table S2 (Additional file 1), the most prevalent ACE was witnessing violent acts in their neighborhood or near the school, followed by parents separated or divorced and lack of support, love, or protection. As for the prosocial behavior, 2872 students had a mean score of 7.12 (SD: 1.75, median: 7.0, IQR: 6–8). Out of those, 1848 (64.35%) were classified as close to the average; 503 (17.51%) as slightly lowered; 342 (11.91%) as low; and 179 (6.23%) as very low. Finally, the SRQ-20 showed a high proportion of the sample had seven or more depression symptoms (19.00%).

Tables 1 and 2 show association tests between our main variables (ACEs in Table 1, and prosocial behavior in Table 2) and the other mental health indicators. Exposure to ACEs was associated to all tested variables, with higher levels of exposure to adversity among females. The adolescents exposed to four or more ACEs obtained lower scores of well-being and satisfaction with life, higher levels of internalizing and externalizing symptoms, and higher prevalence of self-injurious behaviors. Similarly, prosocial behavior was associated with most variables, except the SDQ subscale on emotional problems and the prevalence of suicide attempts, and lower levels of prosocial behavior were found among men. Those with low prosocial behavior (score 0–5) had significantly lower scores of well-being, satisfaction with life and higher prevalence of self-injury.

**Table 1:** Association between adverse childhood experiences, demographics, and mental health indicators

Variable	*n* ^a^	ACEs < 4	ACEs ≥ 4	Test^b^	*p*-value
Female (%)	2912	51.07	59.25	χ² =18.34	< 0.001
Age (mean, SD)	2724	13.68 (4.20)	14.03 (1.84)	Z= − 4.662	< 0.001
Higher grades 9–11 (%)	2910	39.22	46.64	χ² =19.41	0.002
WHO-5 (mean, SD)	2859	18.00 (4.20)	15.05 (4.84)	Z = 15.999	< 0.001
SWLS score (mean, SD)	2873	26.24 (6.40)	23.17 (6.05)	Z = 14.687	< 0.001
SWLS adjusted score (mean, SD)	2873	3.74 (0.91)	3.31 (0.86)	Z = 14.687	< 0.001
Family APGAR (mean, SD)	2855	16.53 (3.63)	13.27 (4.50)	Z = 19.804	< 0.001
SDQ—total difficulties (mean, SD)	2662	10.60 (5.09)	14.48 (5.30)	Z= − 18.064	< 0.001
SDQ—emotional symptoms (mean, SD)	2832	2.86 (2.11)	4.35 (2.37)	Z= − 16.129	< 0.001
SDQ—conduct problems (mean, SD)	2845	2.02 (1.59)	2.90 (1.76)	Z= − 13.579	< 0.001
SDQ—hyperactivity (mean, SD)	2847	3.60 (2.00)	4.32 (2.02)	Z = − 9.022	< 0.001
SDQ—peer problems (mean, SD)	2849	2.20 (1.66)	2.92 (1.81)	Z= − 10.531	< 0.001
SDQ internalizing (mean, SD)	2773	5.05 (3.09)	7.28 (3.41)	Z= − 16.860	< 0.001
SDQ externalizing (mean, SD)	2783	5.62 (3.04)	7.22 (3.15)	Z= − 12.606	< 0.001
SRQ-20—total score (mean, SD)	2677	3.84 (3.66)	7.80 (4.63)	Z= − 22.329	< 0.001
SRQ-20—anxiety (mean, SD)	2792	1.82 (1.96)	3.57 (2.45)	Z= − 19.631	< 0.001
SRQ-20—depression (mean, SD)	2752	2.56 (2.56)	5.40 (3.30)	Z= − 22.735	< 0.001
Self-injury behaviors—any (%)	2918	19.01	49.13	χ² =293.40	< 0.001
Self-injury behaviors—non-suicidal (%)	2910	18.20	46.69	χ² =269.14	< 0.001
Self-injury behaviors—suicidal (%)	2899	3.74	18.87	χ² =182.55	< 0.001

Family APGAR, Family adaptability, partnership, growth, affection, and resolve. SDQ, Strengths and difficulties questionnaire. SRQ-20, Self-reporting questionnaire, 20-item version. SWLS, Satisfaction with life scale. WHO-5, World Health Organization-five well-being index

^a^Each question has a different total number of respondents as participants could answer freely

^b^ Z score corresponds to a Mann–Whitney test and χ² corresponds to a Pearson’s χ² test

**Table 2:** Association between prosocial behavior, demographics, and mental health indicators

Variable	*n* ^a^	Prosocial behavior		Test^b^	*p*-value
		Score 6–10	Score 0–5		
Female (%)	2868	58.85	45.25	χ² =48.98	< 0.001
Age (mean, SD)	2684	13.74 (1.83)	13.98 (1.81)	Z= − 3.434	< 0.001
Grades 9–11 (%)	2866	40.76	44.66	χ² = 4.11	0.043
WHO-5 (mean, SD)	2817	17.53 (4.44)	15.73 (4.86)	Z = 9.72	< 0.001
SWLS score (mean, SD)	2832	25.79 (6.40)	23.89 (6.31)	Z = 8.642	< 0.001
SWLS adjusted score (mean, SD)	2832	3.68 (0.91)	3.41 (0.91)	Z = 8.64	< 0.001
Family APGAR (mean, SD)	2815	15.87 (4.03)	14.32 (4.53)	Z = 9.289	< 0.001
SDQ—total difficulties (mean, SD)	2638	11.38 (5.33)	13.34 (5.56)	Z= − 8.46	< 0.001
SDQ—emotional symptoms (mean, SD)	2799	3.43 (2.35)	3.40 (2.29)	Z = 0.126	0.8994
SDQ—conduct problems (mean, SD)	2805	2.08(1.61)	2.81 (1.77)	Z= − 10.76	< 0.001
SDQ—hyperactivity (mean, SD)	2807	3.67 (2.01)	4.24 (2.04)	Z= − 7.059	< 0.001
SDQ—peer problems (mean, SD)	2808	2.23 (1.67)	2.90 (1.81)	Z= − 9.87	< 0.001
SDQ internalizing (mean, SD)	2743	5.66 (3.33)	6.31 (3.46)	Z= − 4.758	< 0.001
SDQ externalizing (mean, SD)	2746	5.74 (3.06)	7.06 (3.20)	Z = − 10.162	< 0.001
SRQ-20—total score (mean, SD)	2638	5.07 (4.38)	5.74 (4.61)	Z= − 3.736	< 0.001
SRQ-20—anxiety (mean, SD)	2752	2.37 (2.28)	2.66 (2.36)	Z= − 3.39	< 0.001
SRQ-20—depression (mean, SD)	2712	3.44 (3.09)	3.93 (3.28)	Z= − 3.871	< 0.001
Self-injury behaviors—any (%)	2872	28.52	32.71	χ² = 5.53	0.019
Self-injury behaviors—non-suicidal (%)	2866	27.23	31.11	χ² = 4.82	0.028
Self-injury behaviors—suicidal (%)	2855	8.91	9.96	χ² = 0.86	0.354

Family APGAR, Family adaptability, partnership, growth, affection, and resolve. SDQ, Strengths and difficulties questionnaire. SRQ-20, Self-reporting questionnaire, 20-item version. SWLS, Satisfaction with life scale. WHO-5, World Health Organization-five well-being index

^a^Each question has a different total number of respondents as participants could answer freely

^b^ Z score corresponds to a Mann–Whitney test and χ² corresponds to a Pearson’s χ² test

The Spearman correlation coefficient between ACEs and prosocial behavior was − 0.02 (*p*-value = 1.000), so they were not associated in this sample. The logistic regression models (Table 3) found a significant association of a positive depression screening with ACEs (model 1, OR 2.21; 95% CI 1.67–2.94), but not with prosocial behavior (model 2, OR 0.97, 95% CI 0.73–1.28). Besides that, the association between ACEs and depressive symptoms was not modified when including prosocial behavior in the model (model 3), and the test for their interaction did not show statistically significant results (data not shown in Table, OR 0.93, 95% CI 0.54–1.63).

**Table 3:** Logistic regression models showing the association of selected variables with a positive depression screening

Variable in model	Model 1^a^	Model 2^a^	Model 3^b^
	OR (CI 95%)	OR (CI 95%)	OR (CI 95%)
Female	2.77* (2.10–3.66)	2.74* (2.07–3.61)	2.74* (2.07–3.63)
Well-being^b^: worrisome versus otherwise	4.03* (3.08–5.27)	4.03* (3.08–5.27)	3.96* (3.02–5.20)
Satisfaction with life^c^: dissatisfied versus otherwise	2.05* (1.53–2.74)	2.20* (1.64–2.97)	2.10* (1.56–2.83)
Family functioning^d^: moderate-to-severe dysfunction versus otherwise	1.75* (1.32–2.32)	2.00* (1.52–2.64)	1.72* (1.29–2.28)
General psychopathology^e^: high to very high scores versus others	4.74* (3.60–6.24)	5.12* (3.90–6.72)	4.66* (3.53–6.14)
Self-injury: any versus none	2.58* (1.97–3.40)	3.00* (2.30–3.93)	2.61* (1.98–3.44)
Adverse childhood experiences: ≥4 versus ≤ 3	2.21* (1.67–2.94)		2.20* (1.66–2.93)
Prosocial behavior^f^: low versus otherwise		0.97 (0.73–1.28)	0.99 (0.75–1.31)
Constant	0.009* (0.006–0.013)	0.012* (0.009–0.018)	0.010* (0.007–0.014)
Observations	2,417	2,399	2,399
Chi-square test for model	549.2*	533.5*	548.2*
Pseudo-R-squared	0.379	0.364	0.377

Self-reporting questionnaire, depression subscale ≥ 7 points

^a^All models had depression as the outcome variable, and the rows show the Odds Ratio (OR) for each independent variable included in the model, and its 95% Confidence Interval (CI95%) computed with robust standard errors

^b^World Health Organization-Five Well-Being Index (WHO-5): a total score of < 13 or a score ≤ 1 in any item indicates poor wellbeing

^c^Satisfaction With Life Scale (SWLS): dissatisfied (10–14), and very dissatisfied (5–9)

^d^Family Adaptability, Partnership, Growth, Affection, and Resolve (Family APGAR): total scores ranging from 0 to 20, 0–9 indicate severe dysfunction; 10–13, moderate dysfunction; 14–17, mild dysfunction

^e^Strengths and Difficulties Questionnaire (SDQ)—Difficulties scores: The scores for total difficulties are ranked as close to the average (0–14), slightly raised (15–17), high (18, 19) and very high (20–40)

^f^Strengths and Difficulties Questionnaire (SDQ) prosocial behavior subscale: total score of 5 indicates low prosocial behavior and 0–4, very low

*p < 0.01

These models adjusted the associations for several confounding variables that showed association with ACEs and prosocial behaviors (previously described), and with depression scores (data not shown). Although all variables shown in models have a statistically significant association, some of them have a larger effect size; well-being and general psychopathology have the strongest associations with a positive depression screening. As there is correlation between the variables included in the model, we tested for multicollinearity, and found every variable has a low VIF (data not shown, all VIF values were between 1.36 and 1.93).

## Discussion

This study aimed to identify the relationship between ACEs, prosocial behavior, and depression in an adolescent population from urban schools in low-resourced neighborhoods. First, we identified that more than 35% of the adolescents had been exposed to four or more ACEs, and almost one out of every five teenagers were classified as having a low or very low results in prosocial behavior. These were alarming results; they mean a huge proportion of adolescents living under similar conditions are at high risk of psychopathology and other serious outcomes. Even so, they do not mean adolescents under vulnerable conditions are unique to the Colombian population. Similarly, in a Brazilian study, 33.3% of the 15–19-year-old high school students reported four or more ACEs [40], and 72% of adolescents aged 10–16 years in Malawi reported the same [41]. As for the levels of prosocial behavior found in our sample, they are also comparable to those found in similar studies. For example, in a study from Honduras, the authors found a mean score of 7.93 (SD 1.91) in the prosocial behavior subscale of parent-version SDQ [42], and authors in China reported a mean score of 7.24 (SD 2.19) using the same instrument [43]. The concordance in ACEs prevalence and prosocial behavior levels with other studies means our results could be valid in adolescents living under different conditions, even when the questions asked were not the same for ACEs and the prosocial subscale had a low internal consistency.

Second, ACEs were significantly associated with a positive depression screening when controlling for confounding variables (well-being, satisfaction with life, family functioning). A similar association has been documented, Kidman et al. identified that adolescents exposed to 8 or more ACEs were more likely to report depression (three times the odds) and post-traumatic stress disorder (four times the odds) than those exposed to 0–3 ACEs [41]. Moreover, Sahle et al. found a two-fold increase of suicidality, anxiety disorders, depression, and internalizing disorders [44]. Specifically on the topic of suicidality, others found a three-fold increase in rates of suicidal ideation and suicide attempts for adults exposed to three or more ACEs [45]. In Colombia, these results mean trauma-informed practices should be widespread (not focused on certain children); school mental health systems should be in place to care for children who are at a high risk of developing depression and other serious outcomes.

Third, low prosocial behavior was not associated with ACEs nor depression and did not significantly modify ACEs association with depression. Although prosocial behavior has not been as extensively evaluated as ACEs, other authors report and expected association of higher prosocial behaviors with a lower degree of psychopathology [46]. Contrary to our findings, Eli et al. found a statistically significant association between high levels of depression and low levels of both prosocial behavior and resilience [47]. However, this study did not measure/control confounding variables beyond some demographics and the three main variables. There are other studies that identified a statistically significant association between child maltreatment and prosocial behavior, and empathy and gratitude as significant mediators in that association [48]. Nevertheless, that study did not measure psychopathology or any other variables besides demographics and the aforementioned.

Possible explanations for the findings in this study are: The lack of association between exposure to adversity and prosocial behavior; a problem with the measurement of those variables in this sample; and the presence of other unidentified mediators that could serve as protective factors from adversity. We were able to identify at least two other studies that measured both ACEs, prosocial behavior and psychopathology. Bevilacqua et al. evaluated the association between ACEs, psychopathology and prosocial behavior [14], using the parent-version of the SDQ at ages 3, 5, 7, 11 and 14. The authors found that a higher number of ACEs predicted worse mental health and prosocial outcomes, which were evident by age 3 and persisted until adolescence [14]. There is another study that evaluated aggressive behavior and prosocial skills in children exposed to intimate partner violence through preschool and early-school years; their results indicated that there was a cross-domain relation between aggressive behavior problems and prosocial skills, exacerbated by early intimate partner violence exposure [49]. Preschool-age aggressive behavior negatively influenced prosocial skills development during the early school years. Moreover, preschool-age intimate partner violence exposure was linked to decreased early school-age prosocial skills and increased aggressive behavior problems [49]. We do not think there were measurement biases in our study because our findings are quite like other authors’ reports in the prevalence of early adversity and the levels of prosocial behavior. It may be possible that differences are due to the use of different instruments for prosociality as some of them could grasp a more complete picture of the underlying construct.

Exposure to early adversity plays a key role in depression and other mental health outcomes among adolescent population, and its effects may not be alleviated by the presence or positive mental health constructs like prosocial behavior. New studies are struggling to understand the protective effect of benevolent childhood experiences, resilience, and many other positive psychology constructs or protective factors. Those studies need to be conducted in a way that facilitates a better understanding of what constructs are underlying traits, events, neurobiological states, and outcomes; for example, depending on the framework of a research project, prosociality could be modeled either as a trait to explain differences in depression, or as a variable with bidirectional association with depressive symptoms throughout time which in turn explains satisfaction with life as an outcome in the long term. When generating models for understanding the complex phenomenon of mental health, these findings support that they should consider multiple variables and their interactions, even more so in the developmental process of children and adolescents.

As with any cross-sectional study, our project has some limitations. The measurement of prosocial behavior was performed with a subscale of the SDQ (a screening instrument for general psychopathology) and we obtained a low internal consistency; there are other scales that are specifically designed to measure that construct, and they could be considered more thorough, such as the Prosociality Scale [50]. Its reduced version has been used in Colombian [51], Argentinian [52] and Japanese [53] adolescents; however, it is designed for young adults [54]. There is a widespread need for locally validated measures that allow comparison between populations using the same instruments. Besides, this study has weaknesses inherent to cross-sectional studies, such as the inability to measure incidence, difficulty in making causal inferences, associations that are difficult to interpret, and susceptibility to non-response from the participants.

Nonetheless, our response rate was remarkably high in comparison to similar studies on school mental health, and a high response rate allowed us to reduce errors in the estimation of the associations found. Recall bias might be another issue as we asked retrospectively about ACEs using questions adapted from standardized measures available in other studies [27]. This is an inevitable limitation, but longitudinal follow-up and assessment of ACEs would be costly in many ways as well. Our instrument captured 19 different ACEs and was reviewed by experts and members of the schools’ communities to ensure it would be appropriate for adolescents. We adapted questions from multiple questionnaires to assess as many ACEs as possible. There are many instruments available as adversity measures, but few of them have been tested in Colombian adolescents [27, 28].

## Conclusions

ACEs were significantly associated with a positive depression screening when controlling for prosocial behavior and confounding variables such as well-being, satisfaction with life and family functioning. Thus, implementation of trauma-informed mental health promotion strategies at schools, primary care and community mental health centers, is cornerstone to improve the well-being and positive mental health outcomes during adolescence. Furthermore, acknowledgement of the associations -or their absence- between these variables is key when planning how to prevent mental health illnesses in this population and settle regular follow-ups for those at higher risk, as teens exposed to multiple ACEs.

Models generated within the ecological framework could help to better understand the interaction of factors at different levels as well as to help design and carry out effective programs for prevention of mental disorders. Trauma-informed school mental health should not be understood as the performance of universal screening, that approach might be impossible for some schools and difficult to handle for many more of them. As some variables are not modifiable, such as age, sex, and exposure to ACEs, interventions could be directed to improve adolescents’ well-being and health by enhancing factors like satisfaction with life, family functionality, and prosocial behavior. Such an approach might seem logical and useful, but it may not be the most efficient way to address the problem.

Participant charter schools in this study could prioritize their needs and focus their resources on an optimal way for different profiles of their students. Selective and indicated prevention strategies or interventions could help some of the children, but they would be costly for most schools in Colombia. Those strategies should be prioritized within the healthcare system, but there is a burden the healthcare system cannot handle and early diagnosis without treatment is only leading to a larger mental health gap, even more so under inequity conditions. Another strategy to overcome this gap is community participation for the development of interventions that could promote mental health in a universal manner, and positive psychology constructs serving as protective factors and mental health determinants should be the priority of such efforts in research and practice.

Finally, in this sample, prosocial behavior was not associated with ACEs nor depressive symptoms and did not significantly modify the association between ACEs and depression. Intervening prosociality in adolescents exposed to multiple ACEs might not be enough to prevent or treat depression, as they may need trauma-focused interventions that address other issues. On the contrary, intervening prosociality in adolescents with low exposure to ACEs might be useful to increase well-being and other positive mental health outcomes. It should be noted that the design and implementation of these interventions requires further research and a multidisciplinary approach, which includes participation of the education sector.

### Electronic supplementary material

Below is the link to the electronic supplementary material.

Additional file 1.

## Data Availability

The datasets used and/or analyzed during the current study are available from the corresponding author on reasonable request.

## Abbreviations

ACEs: Adverse childhood experiences
SD: Standard Deviation
IQR: Interquartile range
SDQ: Strengths and Difficulties Questionnaire
e.g.: Exempli gratia
WHO-5: World Health Organization-five well-being index
SWLS: Satisfaction with life scale
Family APGAR: Family adaptability, partnership, growth, affection, and resolve
SRQ-20: Self-reporting questionnaire, 20-item version
NMHS: National Mental Health Survey
OR: Odds ratio

## References

[1] Davis JP, Ports KA, Basile KC, Espelage DL, David-Ferdon CF (2019). Understanding the buffering effects of protective factors on the relationship between adverse childhood experiences and teen dating violence perpetration. J Youth Adolesc.

[2] Jones CM, Merrick MT, Houry DE (2020). Identifying and preventing adverse childhood experiences: implications for clinical practice. JAMA.

[3] McLaughlin KA (2016). Future directions in childhood adversity and youth psychopathology. J Clin Child Adolesc Psychol.

[4] Merrick MT, Ford DC, Ports KA, Guinn AS, Chen J, Klevens J (2019). Vital signs: estimated proportion of adult health problems attributable to adverse childhood experiences and implications for prevention—25 states, 2015–2017. MMWR Morb Mortal Wkly Rep.

[5] Hughes K, Bellis MA, Hardcastle KA, Sethi D, Butchart A, Mikton C (2017). The effect of multiple adverse childhood experiences on health: a systematic review and meta-analysis. Lancet Public Health.

[6] Chang X, Jiang X, Mkandarwire T, Shen M (2019). Associations between adverse childhood experiences and health outcomes in adults aged 18–59 years. PLoS One.

[7] Schilling EA, Aseltine RH, Gore S (2007). Adverse childhood experiences and mental health in young adults: a longitudinal survey. BMC Public Health.

[8] Merrick MT, Ford DC, Ports KA, Guinn AS (2018). Prevalence of adverse childhood experiences from the 2011–2014 behavioral risk factor surveillance system in 23 states. JAMA Pediatr.

[9] Devries K, Merrill KG, Knight L, Bott S, Guedes A, Butron-Riveros B (2019). Violence against children in Latin America and the Caribbean: what do available data reveal about prevalence and perpetrators?. Pan Am J Public Health.

[10] Castillo Martínez A, Cleves Luna D, García Cifuentes ÁM, Laverde Martínez L, Medina Medina V, Cortés Ruiz H (2017). Experiencias adversas de la infancia en una muestra de pacientes con enfermedad crónica en Cali-Colombia. Med UPB.

[11] Bautista Bautista NE, Cuello Royert C, Moreno MY, Camargo L, Gaviria Uribe A, Correa LF, et al. Observatorio Nacional de Salud Mental, ONSM Colombia. In: Mental SdENTGFGIplS, editor. Colombia: Ministerio de Salud y Protección social; 2017. p. 47

[12] Chapman DP, Whitfield CL, Felitti VJ, Dube SR, Edwards VJ, Anda RF (2004). Adverse childhood experiences and the risk of depressive disorders in adulthood. J Affect Disord.

[13] Bjorkenstam E, Hjern A, Mittendorfer-Rutz E, Vinnerljung B, Hallqvist J, Ljung R (2013). Multi-exposure and clustering of adverse childhood experiences, socioeconomic differences and psychotropic medication in young adults. PLoS One.

[14] Bevilacqua L, Kelly Y, Heilmann A, Priest N, Lacey RE (2021). Adverse childhood experiences and trajectories of internalizing, externalizing, and prosocial behaviors from childhood to adolescence. Child Abus Negl.

[15] Carvalho F, Maciel L, Basto-Pereira M (2020). Two sides of child maltreatment: from psychopathic traits to altruistic attitudes inhibition. J Child Adolesc Trauma.

[16] Gomis-Pomares A, Villanueva L (2020). The effect of adverse childhood experiences on deviant and altruistic behavior during emerging adulthood. Psicothema.

[17] Rohner SL, Salas Castillo AN, Carr A, Thoma MV (2022). Childhood adversity and later life prosocial behavior: a qualitative comparative study of Irish older adult survivors. Front Psychol.

[18] Gupita D, Thapliyal G (2015). A study of prosocial behaviour and self concept of adolescents. i-manager’s. J Educ Psychol.

[19] Hastings P, Utendale WT, Sullivan C. The socialization of prosocial development. Handb Social Theory Res. 2007;20:638–64.

[20] Aknin LB, Van de Vondervoort JW, Hamlin JK (2018). Positive feelings reward and promote prosocial behavior. Curr Opin Psychol.

[21] Caprara GV, Barbaranelli C, Pastorelli C, Bandura A, Zimbardo PG (2000). Prosocial foundations of children’s academic achievement. Psychol Sci.

[22] Snippe E, Jeronimus BF, Aan Het Rot M, Bos EH, de Jonge P, Wichers M (2018). The reciprocity of prosocial behavior and positive affect in daily life. J Personal.

[23] Padilla-Walker LM, Memmott-Elison MK, Coyne SM (2018). Associations between prosocial and problem behavior from early to late adolescence. J Youth Adolesc.

[24] Alarcón G, Forbes EE (2017). Prosocial Behavior and depression: a case for developmental gender differences. Curr Behav Neurosci Rep.

[25] Bernal R, Pulido X, Sanchez F, Lina S. Decisiones de vida de los jóvenes en Bogotá: pobreza, habilidades o comportamientos de riesgo?. In: Novella R, Repetto A, Robino C, Rucci G, editors. Millennials en América Latina y el Caribe: trabajar o estudiar?. Chile; 2018. p. 175–206.

[26] Ministerio de Salud (Department of Health) MdCDoS. Base de datos Encuesta Nacional de Salud Mental 2015 (National Mental Health Survey 2015 dataset). 2015.

[27] Bethell CD, Carle A, Hudziak J, Gombojav N, Powers K, Wade R (2017). Methods to assess adverse childhood experiences of children and families: toward approaches to promote child well-being in policy and practice. Acad Pediatr.

[28] Barrios M, Riaño SV, Villa YM, Martinez OP, Aragon YA, Meyer S (2017). Correlation between growth and adverse childhood experiences in vulnerable children in Bogotá, Colombia. J Child Adolesc Trauma.

[29] Goodman R, Meltzer H, Bailey V (1998). The strengths and difficulties questionnaire: a pilot study on the validity of the self-report version. Eur Child Adolesc Psychiatry.

[30] Youth In Mind. Explanation of how scoring is done for SDQs for 4–18 year olds, as completed by parents, teachers or youths 2016. Available from: https://sdqinfo.org/py/sdqinfo/c0.py.

[31] World Health Organization. Wellbeing measures in primary health care/The DEPCARE project. In: Europe, WROf, editor. Stockholm: World Health Organization; 1998.

[32] Campo-Arias A, Miranda-Tapia GA, Cogollo Z, Herazo E (2015). Reproducibility of the well-being index (WHO-5 WBI) among adolescent students. Revist Cient Salud Uninorte.

[33] Allgaier A-K, Pietsch K, Frühe B, Prast E, Sigl-Glöckner J, Schulte-Körne G (2012). Depression in pediatric care: is the WHO-five well-being index a valid screening instrument for children and adolescents?. Gen Hosp Psychiatry.

[34] Vinaccia Alpi S, Parada N, Quiceno JM, Riveros Munévar F, Vera Maldonado LA (2019). Satisfaction with life scale (SWLS): validity, reliability and assessment analysis in college students from Bogotá (col) as sample. Psicogente.

[35] Vázquez C, Duque A, Hervás G (2013). Satisfaction with life scale in a representative sample of Spanish adults: validation and normative data. Span J Psychol.

[36] Forero Ariza LM, Avendaño Durán MC, Duarte Cubillos ZJ, Campo-Arias A (2006). Consistencia interna y análisis de factores de la escala APGAR para Evaluar El funcionamiento familiar en estudiantes de básica secundaria. Revist Colombiana de Psiquiatría.

[37] Smilkstein G, The Family APGAR (1978). A proposal for a family function test and its use by physicians. J Fam Pract.

[38] Beusenberg M, Orley JH, World Health Organization. A user’s guide to the self reporting questionnaire (SRQ/compiled by M. Beusenberg and J. Orley. In: Health DoM, editor. World Health Organization; 1994.

[39] Suarez DE, Cardozo AC, Ellmer D, Trujillo EM. Short report: cross sectional comparison of anxiety and depression symptoms in medical students and the general population in Colombia. Psychol Health Med. 2020:1–6. 10.1080/13548506.2020.1757130 32314943

[40] Reisen A, Viana MC, Dos Santos Neto ET (2019). Adverse childhood experiences and bullying in late adolescence in a metropolitan region of Brazil. Child Abuse Negl.

[41] Kidman R, Piccolo LR, Kohler HP (2020). Adverse childhood experiences: prevalence and association with adolescent health in Malawi. Am J Prev Med.

[42] Harry ML, Acevedo J, Crea TM (2019). Assessing the factor structure of the Spanish language parent strengths and difficulties questionnaire (SDQ) in Honduras. PLoS One.

[43] Liu Q, Zhou Y, Xie X, Xue Q, Zhu K, Wan Z (2021). The prevalence of behavioral problems among school-aged children in home quarantine during the COVID-19 pandemic in China. J Affect Disord.

[44] Sahle BW, Reavley NJ, Li W, Morgan AJ, Yap MBH, Reupert A et al. The association between adverse childhood experiences and common mental disorders and suicidality: an umbrella review of systematic reviews and meta-analyses. Eur Child Adolesc Psychiatry. 2021. 10.1007/s00787-021-01745-2 33638709

[45] Thompson MP, Kingree JB, Lamis D (2019). Associations of adverse childhood experiences and suicidal behaviors in adulthood in a U.S. nationally representative sample. Child Care Health Dev.

[46] Memmott-Elison MK, Holmgren HG, Padilla-Walker LM, Hawkins AJ (2020). Associations between prosocial behavior, externalizing behaviors, and internalizing symptoms during adolescence: a meta-analysis. J Adolesc.

[47] Eli B, Zhou Y, Liang Y, Cheng J, Wang J, Huang C (2021). Depression in children and adolescents on the Qinghai-Tibet Plateau: associations with resilience and prosocial behavior. Int J Environ Res Public Health.

[48] Yu G, Li S, Zhao F (2020). Childhood maltreatment and prosocial behavior among Chinese adolescents: roles of empathy and gratitude. Child Abuse Negl.

[49] Holmes MR, Voith LA, Gromoske AN (2015). Lasting effect of intimate partner violence exposure during preschool on aggressive behavior and prosocial skills. J Interpers Violence.

[50] Caprara GV, Steca P, Zelli A, Capanna C (2005). A new scale for measuring adults’ prosocialness. Eur J Psychol Assess.

[51] Luengo Kanacri BP, Eisenberg N, Thartori E, Pastorelli C, Uribe Tirado LM, Gerbino M (2017). Longitudinal relations among positivity, perceived positive school climate, and prosocial behavior in Colombian adolescents. Child Dev.

[52] Rodriguez L, Mesurado B, Oñate M, Guerra P, Menghi MS (2017). Adaptación de la escala de prosocialidad de caprara en Adolescentes Argentinos. Revista Evaluar.

[53] Murakami T, Nishimura T, Sakurai S (2016). Prosocial behavior toward family, friends, and strangers: development of a prosocial behavior scale focused on the recipient of the behavior. Jpn J Educ Psychol.

[54] Martí-Vilar M, Merino-Soto C, Rodriguez LM (2020). Measurement invariance of the prosocial behavior scale in three hispanic countries (Argentina, Spain, and Peru). Front Psychol.

