# Supplementary material for: Early adversity and prosocial behavior in adolescents from Bogotá: a cross-sectional study
Source: Child Adolesc Psychiatry Ment Health. 2024 Jul 8;18:81. doi: 10.1186/s13034-024-00768-2 (PMC11232159; doi:10.1186/s13034-024-00768-2)
Supplement: Supplementary file 1 — Additional file 1. [file 13034_2024_768_MOESM1_ESM.docx]

**Box 1. Questionnaire about Adverse Childhood Experiences.**

| 1. Have you ever lived with someone who suffers depression (intense sadness for a long period of time), or who has a mental illness or has attempted suicide? 2. Have you ever lived with someone who has problems due to the use of alcohol or other substances (cannabis, cocaine, or any other)? 3. Have you ever lived with someone who has been in jail? 4. Have the people you live with hurt or threatened to hurt each other? 5. Have you ever been treated badly because of personal characteristics such as the color of your skin, your sexual orientation, where you come from, a disability, your religion, or any other reason? 6. Have you ever lacked food, clothing, or a place to live? 7. Have you ever felt lack of support, love, or protection? 8. Are your parents separated or divorced? 9. Have you been a victim of forced displacement, or have you witnessed violent acts related to the armed conflict? 10. Has the person who cared for you -like your father, mother or someone who replaces that role- died? 11. Have you suffered a serious illness, or have you had a surgery that has put your life in danger? 12. Have you seen or heard violent acts in your neighborhood or near your school? 13. Has someone you live with insulted you, treated you badly, or acted in a way that made you afraid of being physically harmed? 14. Have you been physically abused (slapping, hitting) by someone you live with? 15. Have you ever experienced bullying or intimidation at school (attacks or repeated abuse by one or more classmates)? 16. Have you ever experienced verbal or physical abuse or threats from your partner, boyfriend or girlfriend? 17. Have you ever been under custody by the child protective services? 18. Have you ever been arrested or imprisoned? 19. Has someone touched your intimate parts or asked you to touch their intimate parts against your will or has made you feel uncomfortable? |
| --- |

**Table S1. Distribution of mental health indicators for the whole sample.**

| **Scale** | ***n*^a^** | **Mean (SD)** | **Normality test^b^** | **Cronbach’s alpha** |
| --- | --- | --- | --- | --- |
| **WHO-5** | 2859 | 16.89 (4.67) | 9.41 | α = 0.81 |
| **SWLS** | 2873 | 25.09 (6.44) | 11.31 | α = 0.82 |
| **SWLS adjusted** | 2873 | 3.58 (0.92) | 11.31 | - |
| **Family APGAR** | 2855 | 15.32 (4.27) | 10.62 | α = 0.89 |
| **SDQ – prosocial subscale** | 2872 | 7.13 (1.75) | 5.24 | α = 0.58 |
| **SDQ – total difficulties** | 2662 | 12.06 (5.49) | 7.14 | α = 0.77 |
| **SDQ – emotional symptoms** | 2832 | 3.42 (2.32) | 7.07 | α = 0.70 |
| **SDQ – conduct problems** | 2845 | 2.35 (1.71) | 8.45 | α = 0.53 |
| **SDQ – hyperactivity** | 2847 | 3.87 (2.04) | 3.17 | α = 0.61 |
| **SDQ – peer problems** | 2849 | 2.47 (1.75) | 8.67 | α = 0.48 |
| **SDQ – internalizing** | 2773 | 5.89 (3.39) | 8.77 | α = 0.70 |
| **SDQ - externalizing** | 2783 | 6.22 (3.18) | 5.74 | α = 0.69 |
| **SRQ-20 – total score** | 2677 | 5.32 (4.48) | 10.89 | α = 0.86 |
| **SRQ-20 – anxiety** | 2792 | 2.48 (2.31) | 10.02 | α = 0.74 |
| **SRQ-20 – depression** | 2752 | 3.63(3.17) | 10.57 | α = 0.81 |

^a^Each question has a different total number of respondents as participants could answer freely. ^b^All variables showed a p-value <0.001 in the Shapiro – Francia normality test. Family APGAR: Family Adaptability, Partnership, Growth, Affection, and Resolve. SDQ: Strengths and Difficulties Questionnaire. SRQ-20: Self-reporting questionnaire, 20-item version. SWLS: Satisfaction With Life Scale. WHO-5: World Health Organization-Five Well-Being Index.

**Table S2. Prevalence of Adverse Childhood Experiences (most to least prevalent).**

| **Adverse Childhood Experiences** | ***n/N^a^*** | **Prevalence (%)** |
| --- | --- | --- |
| Witnessing violent acts in neighborhood or near school | 1718/2898 | 59.28% |
| Parents separated or divorced | 1178/2896 | 40.68% |
| Lack of support, love, or protection | 1023/2890 | 35.40% |
| Physical abuse | 681/2887 | 23.59% |
| Discrimination | 593/2896 | 20.48% |
| School bullying | 549/2893 | 18.98% |
| Psychological abuse | 536/2891 | 18.54% |
| Lived with someone with substances’ problematic use | 508/2894 | 17.55% |
| Lived with someone with a serious mental illness | 463/2907 | 15.93% |
| Death of a caregiver | 395/2888 | 13.68% |
| Serious illness or life-threatening surgery | 346/2887 | 11.98% |
| Lived with someone who has been in jail | 314/2895 | 10.85% |
| Domestic violence | 226/2894 | 7.83% |
| Sexual abuse | 166/2893 | 5.74% |
| Victim of partner abuse | 94/1865 | 5.04% |
| Child protective services | 139/2888 | 4.81% |
| Poverty | 102/2893 | 3.53% |
| Forced displacement and/or armed conflict exposure | 102/2892 | 3.53% |
| Arrested or imprisoned | 58/2891 | 2.01% |

^a^Each question has a different total number of respondents as participants could answer freely.
